# Representation of Instantaneous and Short-Term Loudness in the Human Cortex

**DOI:** 10.3389/fnins.2016.00183

**Published:** 2016-04-28

**Authors:** Andrew Thwaites, Brian R. Glasberg, Ian Nimmo-Smith, William D. Marslen-Wilson, Brian C. J. Moore

**Affiliations:** ^1^Department of Psychology, University of CambridgeCambridge, UK; ^2^Medical Research Council Cognition and Brain Sciences UnitCambridge, UK

**Keywords:** magnetoencephalography, MNE source space, loudness, sound, perception, information encoding, model expression, entrainment

## Abstract

Acoustic signals pass through numerous transforms in the auditory system before perceptual attributes such as loudness and pitch are derived. However, relatively little is known as to exactly when these transformations happen, and where, cortically or sub-cortically, they occur. In an effort to examine this, we investigated the latencies and locations of cortical entrainment to two transforms predicted by a model of loudness perception for time-varying sounds: the transforms were instantaneous loudness and short-term loudness, where the latter is hypothesized to be derived from the former and therefore should occur later in time. Entrainment of cortical activity was estimated from electro- and magneto-encephalographic (EMEG) activity, recorded while healthy subjects listened to continuous speech. There was entrainment to instantaneous loudness bilaterally at 45, 100, and 165 ms, in Heschl's gyrus, dorsal lateral sulcus, and Heschl's gyrus, respectively. Entrainment to short-term loudness was found in both the dorsal lateral sulcus and superior temporal sulcus at 275 ms. These results suggest that short-term loudness is derived from instantaneous loudness, and that this derivation occurs after processing in sub-cortical structures.

## Introduction

The loudness of a sound corresponds to the subjective impression of its magnitude. While loudness is partly determined by the physical intensity of a sound, it is also strongly affected by frequency content (spectrum) and by fluctuations in the sound over time, as well as by the way that sound is transformed and processed in the auditory system. As sound passes through the outer ear, middle ear, and cochlea, it is subjected to a variety of transformations, including spectral shaping, filtering into multiple frequency channels, and amplitude compression (Moore et al., 1997; Moore, 2012). At later stages in the auditory system, temporal integration occurs, since loudness depends on the time history of the sound, and the loudness of a brief sound increases with increasing duration for durations up to at least 100 ms (Scharf, 1978). Glasberg and Moore (2002) proposed that there are two aspects of loudness for time-varying sounds such as speech and music. The short-term loudness corresponds to the loudness of a short segment of sound such as a single word in speech or a single note in music. The long-term loudness corresponds to the overall loudness of a relatively long segment of sound, such as a whole sentence or a musical phrase. They proposed a model in which transformations and processes that are assumed to occur at relatively peripheral levels in the auditory system (i.e., the outer, middle, and inner ear) are used to construct a quantity called “instantaneous loudness” that is not available to conscious perception. At later stages in the auditory system the neural representation of the instantaneous loudness is transformed into the short-term loudness and long-term loudness, via processes of temporal integration. This leads to the following questions: (1) At what stage or stages in the auditory pathway are the transforms leading to short-term and long-term loudness taking place? (2) Of those transformations taking place in the cortex, where do these transformations take place? Some authors have put the first question differently, by asking: “at what stage or stages along the auditory pathway is sound intensity transformed into its perceptual correlate (i.e., loudness)?” (Behler and Uppenkamp, 2016). In our view, this question is not meaningful, since sound intensity is never directly represented in the auditory pathway. Even at the most peripheral level of auditory neural coding (the auditory nerve), substantial transformations of the sound have already occurred.

While imaging studies suggest that loudness is represented by activation in the cortex (Hall et al., 2001; Langers et al., 2007; Röhl and Uppenkamp, 2012; Giordano et al., 2013), inferring when and where short-term and long-term loudness are constructed is challenging. Looking for evidence in structures earlier in the auditory pathway, such as the inferior colliculus (IC) and medial geniculate body (MGB), is an important first step: findings from Röhl and Uppenkamp (2012) suggest that the loudness may be constructed subsequent to processing in the IC. However, most of these previous studies used only quasi-steady sounds as stimuli (e.g., tones bursts) and they did not distinguish between short-term and long-term loudness.

In this study we focus on the transformation of instantaneous loudness to short-term loudness. The instantaneous loudness is the estimated value of the magnitude of the output of the cochlea after passage through the outer and middle ear, creation of an excitation pattern in the cochlea via processing through an array of bandpass filters (Moore and Glasberg, 1983; Glasberg and Moore, 1990), and application of amplitude compression (Moore et al., 1997). A model for predicting the loudness of steady sounds based on this approach gives accurate predictions of a variety of perceptual data in the literature (e.g., Gässler, 1954; Langhans and Kohlrausch, 1992; see, Moore, 2014, for overview), and forms the basis for the current ANSI standard for calculation of the loudness of steady sounds (The American National Standards Institute,, 2007). The short-term loudness is assumed to be determined at a subsequent stage of auditory processing via a running average of the instantaneous loudness, using an averaging process resembling the operation of an automatic gain control system (Glasberg and Moore, 2002).

We sought evidence of the latencies of the representations of instantaneous loudness and short-term loudness by examining if either of them is “tracked” by cortical current, a phenomenon known as cortical entrainment (Ding and Simon, 2014; Ding et al., 2014). Cortical entrainment to the stimulus magnitude (normally characterized by the Hilbert envelope of the broadband signal) has been found through correlation to electro-encephalographic (EEG), magneto-encephalographic (MEG), and intracranial-EEG data (e.g., Ahissar et al., 2001; Luo and Poeppel, 2007; Aiken and Picton, 2008; Nourski et al., 2009; Kubanek et al., 2013), as well as in studies showing cortical entrainment to the speech envelope after it has been convolved with an impulse response, estimated using the spectro-temporal response function (Aiken and Picton, 2008; Mesgarani et al., 2009; Ding and Simon, 2012; Pasley et al., 2012; Zion Golumbic et al., 2013) or evoked spread spectrum analysis (Lalor et al., 2009; Power et al., 2012). In those cases where it was possible to localize the entrainment, it was normally found in auditory cortex, with a latency of 300 ms or less. More recently, entrainment to more realistic models of sound magnitude, such as instantaneous loudness, were found in Heschl's gyrus (Thwaites et al., 2015), with a latency of 100 ms.

The current study aimed to replicate and refine the findings of Thwaites et al. (2015) using a larger data set and to determine whether there was also entrainment to short-term loudness. Specifically, we aimed to determine: (1) what the latencies were for this entrainment for each aspect of loudness and (2) what were the cortical locations of this entrainment. We also assessed entrainment to the Hilbert envelope for comparison with earlier work, even though the Hilbert envelope takes no account of the filtering by the outer and middle ear, or of the bandpass filtering and compression that occur in the cochlea.

In addition to standard graphic representations, an interactive representation of this study's results can be viewed on the online Kymata Atlas (http://kymata-atlas.org). For easy reference, each model in this paper (referred to as a “function” in Kymata) is assigned a *Kymata ID* [KID].

### Defining candidate models

In order to measure cortical entrainment, some trivial constraints must be imposed on the models that we can test. We can consider any model that takes a time-varying signal as input and produces a time-varying signal as output, with function *f*() characterizing the mechanism by which the information (in this case the acoustic waveform) is transformed before it produces cortical entrainment. Thus, if both input x_1,_…, x_*m*_ and output y_1,_…,y_*n*_ are of duration *t*, the model takes the form:

(1) $$\begin{array}{r} f\left(x_{1},x_{2},x_{3},\ldots ,x_{t}\right)=\left(y_{1},y_{2},y_{3},\ldots ,y_{t}\right) \end{array}$$

where *f*() is bounded both by a set of formal requirements (Davis et al., 1994) and a requirement that y_i_ cannot be dependent on any x_k_ where k > i (this last requirement avoids hypothesizing a non-causal *f*() where a region can express an output before it has the appropriate input).

In the following section, we specify three candidate models two of which are based on progressively more complex transforms of the input signal.

### The candidate models

The two “auditory magnitude” models are characterizations of the transformation between the input acoustic signal (the time-varying sound pressure) and its representation in the auditory system, including the cortex. These models, the instantaneous loudness model and short-term loudness model, represent successive transformations that approximate physiological processing in the peripheral and central auditory system. The latter approximates the perceived momentary magnitude of a sound.

**Figure 1:**
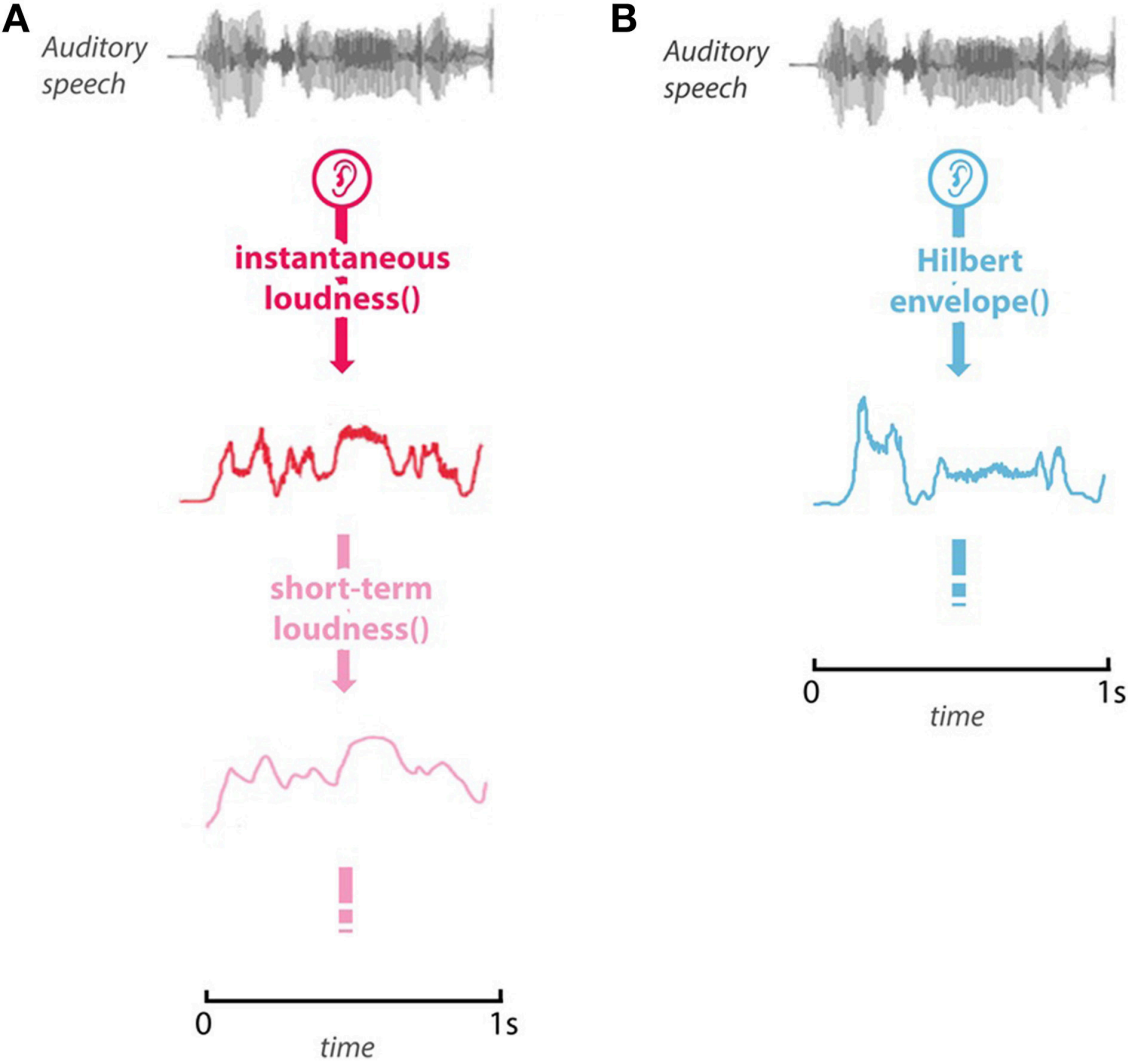
**Example of the stimulus and model predictions. (A)** The predicted *instantaneous loudness* (red), and *short-term loudness* (pink) for the first 1-s of the stimulus. **(B)** The *Hilbert envelope* for the first second of the stimulus.

The third model, the Hilbert envelope model, is also a measure of the time-varying magnitude of a sound, but it is uninformed by physiological processing, and is intended as a naïve comparison model. Examples of the models' predicted activity are shown in Figure 1.

#### Instantaneous loudness (KID: QRLFE)

Moore et al. (1997) and Glasberg and Moore (2002) developed a model for predicting the loudness of sounds based on a series of stages that mimic processes that are known to occur in the auditory system. The first stage is a linear filter to account for the transfer of sound from the source (e.g., a headphone or loudspeaker) to the tympanic membrane. The second stage is a linear filter to account for the transfer of sound pressure from the tympanic membrane through the middle ear to pressure difference across the basilar membrane within the cochlea. The result of this stage is passed through an array of level-dependent bandpass filters, resembling the filters that exist within the cochlea (Glasberg and Moore, 1990). These filters are often called the “auditory filters.” A compressive nonlinearity is applied to the output of each auditory filter, resembling the compression that occurs in the cochlea (Robles and Ruggero, 2001). Finally, the compressed outputs of the filters are combined to give a quantity proportional to loudness. The unit of loudness is the sone.

The compressed outputs of the auditory filters can vary rapidly in time, but the perception of loudness changes more slowly, presumably reflecting a relatively central temporal integration process. The model described by Glasberg and Moore (2002) calculates the compressed outputs of the auditory filters at 1-ms intervals. The time window over which the compressed output of each filter is calculated varies with center frequency, but is always relatively short. The resulting quantity is called the “instantaneous loudness.” It is assumed that a representation of instantaneous loudness exists in the brain, but that it is not accessible to conscious awareness. A subsequent stage, described in the next section, performs a running temporal integration of the instantaneous loudness to give the short-term loudness.

The operation of the model for the calculation of instantaneous loudness can be summarized using an equation that characterizes a combination of temporal and spectral integration:

(2) $$\begin{array}{l} instantaneous\_loudness\left(x,t\right)\\ \;=\int_{\text{min}\left(\text{a}\right)}^{\text{max}\left(\text{a}\right)}\int_{0}^{\tau }|G\left(a,c,x,t-t\prime \right)|dt\prime da \end{array}$$

where *G*(*a,c,x,t*) is the output at time *t* of a filterbank applied to the stimulus *x*, where *a* is the channel number, and *c* is a constant that determines the degree of compression applied to the output of that channel and that varies with signal level. τ is the width of a temporal averaging window.

#### Short-term loudness (KID: B3PU3)

In the model of Glasberg and Moore (2002) it is assumed that a running average of the instantaneous loudness is taken to give the short-term loudness—the momentary impression of the loudness of a sound. The averaging process in the model resembles the operation of an automatic gain control system with separate attack and release times (*T*_*a*_ and *T*_*r*_, respectively). The short-term loudness estimate increases rapidly when the level of a sound suddenly increases, or when a sound is turned on, but the estimate decreases more slowly when the sound level decreases or the sound is turned off. This reflects the fact that the loudness of a sound increases rapidly when the sound is first turned on, but the loudness impression decays more slowly when the sound is turned off.

We define S${}_{n}^{\prime }$ as the running (averaged) short-term estimate of loudness at the time corresponding to the *n*th time frame (updated every 1 ms), S_*n*_ as the calculated instantaneous loudness at the *n*th time frame, and S′_*n*−1_ as the running loudness at the time corresponding to frame *n*−1.

If S_*n*_ > S${}_{n-1}^{\prime }$ (corresponding to an attack, as the instantaneous loudness at frame *n* is greater than the short-term loudness at the previous frame), then

(3) $$\begin{array}{l} S_{n}^{\prime }=\alpha_{a}S_{n}+\left(1-\alpha_{a}\right)S_{n-1}^{\prime }, \end{array}$$

where α_*a*_ is a constant which is related to the attack time *T*_*a*_:

(4) $$\begin{array}{l} \alpha_{a}=1-e^{\frac{T_{i}}{T_{a}}} \end{array}$$

where *T*_*i*_ is the time interval between successive values of the instantaneous loudness (1 ms in this case). If S_*n*_ ≤ S′_*n*−1_ (corresponding to a release, as the instantaneous loudness is less than the short-term loudness), then

(5) $$\begin{array}{l} S_{n}^{\prime }=\alpha_{r}S_{n}+\left(1-\alpha_{r}\right)S_{n-1}^{\prime }, \end{array}$$

where α_*r*_ is a constant which is related to the release time *T*_*r*_:

(6) $$\begin{array}{l} \alpha_{r}=1-e^{\frac{T_{i}}{T_{r}}} \end{array}$$

#### Hilbert envelope (KID: ZDSQ9)

A third model of auditory magnitude is that provided by the Hilbert envelope. This is a very simple model, intended to provide a baseline for comparison with other models that are more perceptually and physiologically plausible.

A convenient method for extracting the envelope of an acoustic signal makes use of the Hilbert transform (Hilbert, 1912). The Hilbert Transform is the sum of the original signal and the signal phase shifted by 90°. The absolute magnitude of the Hilbert transform gives what is called the Hilbert envelope. The Hilbert envelope sampled at 1-ms intervals was used here:

(7) $$\begin{array}{r} hilbert-env\left(x_{1},\ldots ,x_{\text{n}}\right)=abs\left(hilbert\left(x_{1},\ldots ,x_{\text{n}}\right)\right) \end{array}$$

where *hilbert*() returns the complex helical signal of the input, composed of a real and an imaginary component, and *abs*() returns the magnitude of this signal.

### The analysis procedure

**Figure 2:**
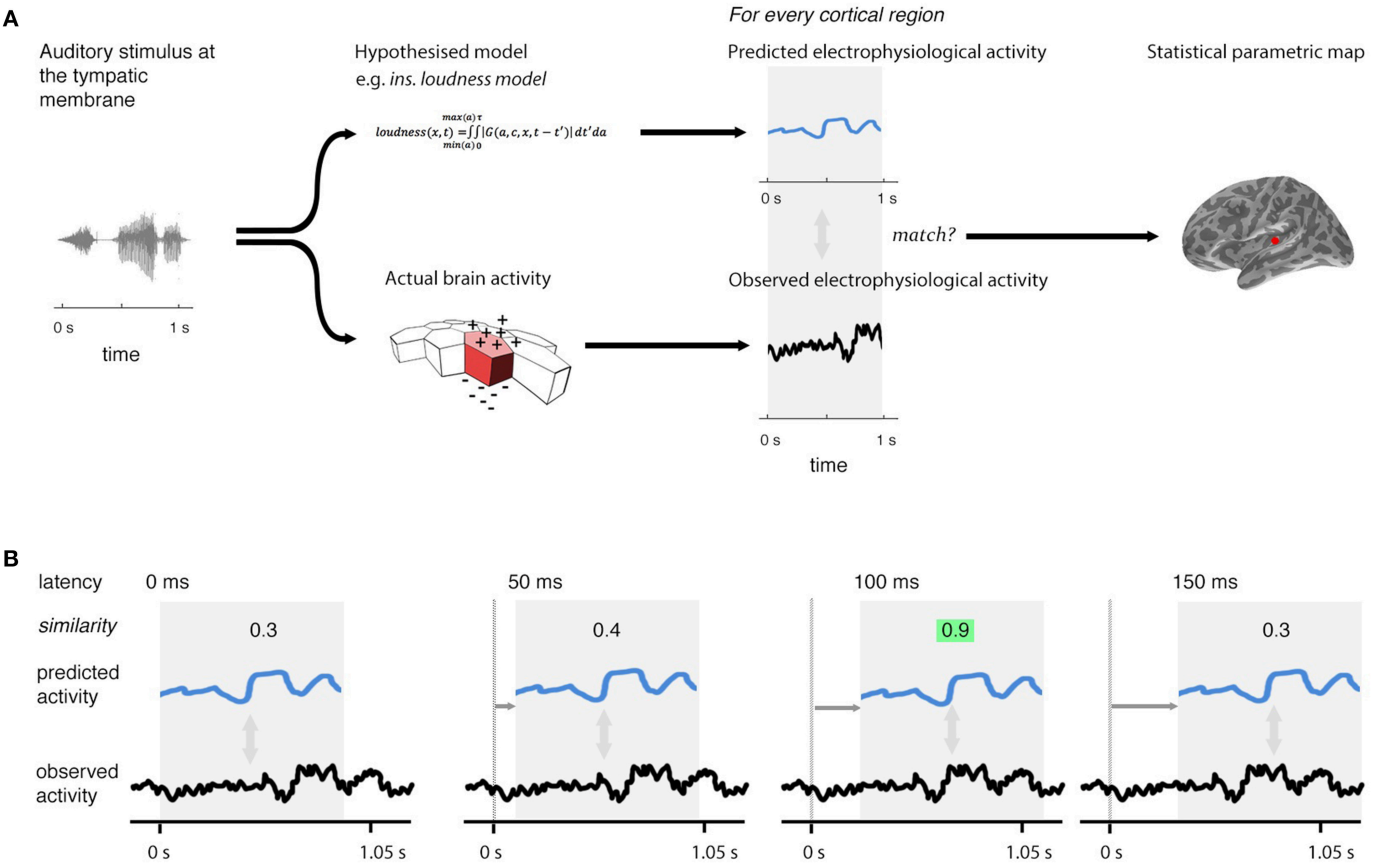
**Technique overview**. First **(A)**, the electrophysiological activity of the brain in response to a given stimulus (measured using EMEG) is matched to the pattern of neural activity predicted by the model being evaluated. Predicted and observed activity are tested for similarity and the resulting statistical parametric map displays the regions (sources) where the match is statistically significant. Second **(B)**, this procedure is repeated at different lags (illustrated here from 0 to 150 ms) between the onset of the observed neural activity and the onset of the predicted output. The similarity is highest at the correct lag (highlighted). This produces a statistical parametric map that changes over time.

The reconstructed distributed source current of the cortex yields the current of 10,242 cortical regions (sources), spaced uniformly over the cortex. The testing procedure involves examining each of these sources, looking for evidence that the current predicted by a model is generating the current observed (Figure 2A). This procedure is repeated at 5-ms intervals (Figure 2B) across a range of time-lags (–200 < *l* < 800 ms), covering the range of plausible latencies (0–800 ms) and a short, pre-stimulation range (–200 to 0 ms) during which we would expect to see no significant match [The 0–800 ms range was chosen because the study of Thwaites et al. (2015) showed little significant expression for instantaneous loudness after a latency of 500 ms; the 5-ms interval step was chosen because it is the smallest value that can be used given current computing constraints]. This produces a statistical parametric map that changes over time as the lag is varied, revealing the evolution of similarity of a given model's predicted behavior with observed behavior over cortical location and time. Evidence of a model's similarity between its predicated behavior and cortical activity is expressed as a *p*-value, which is generated through the match-mismatch technique described in Thwaites et al. (2015), where evidence for similarity is described as significant if the *p*-value is less than a pre-defined value, α^*^. We refer to the observation of significant matches at a specific lag as “model expression.”

Setting α^*^ so that it accurately reflects what is known about the data being tested can be difficult. In the current study, some of the measurements used in the tests are dependent on other measurements (because of spatial and temporal similarities between neighboring sources and lags). However, it is very difficult, if not impossible, to get accurate estimations of, for instance, the spatial dependencies between sources. In the present study, rather than accept assumptions about the dependencies that are hard to justify, we assumed that the data at each source and lag were independent (a “worst case” scenario). As a result, the reader should be aware that the type II error rate is likely to be high, making the reported results “conservative.”

We used an exact formula for the familywise false alarm rate to generate a “corrected” α, α^*^ of ~3 × 10^−13^ (see Thwaites et al., 2015, for the full reasoning); *p*-values greater than this are deemed to be not significant.

The results are presented as *expression plots*, which show the latency at which each of the 10,242 sources for each hemisphere best matched the output of the tested model (marked as a “stem”). The y-axis shows the evidence supporting the match at this latency: if any of the sources have evidence, at their best latency, indicated by a *p*-value lower than α^*^, they are deemed “significant matches” and the stems are colored red, pink or blue, depending on the model.

The expression plots also allow us to chart which models are most likely at a particular source through a model selection procedure, using *p*-values as a proxy for model likelihood. For each model's expression plot we retain only those sources where the *p*-value is lower (has higher likelihood) than for the other two models tested. Accordingly, each plot has fewer than 10,242 sources per hemisphere, but the three plots taken together make up the full complement of 10,242 sources per hemisphere. It is important to note that this model selection procedure does not indicate that any one model is *significantly* better than another for some source. It indicates only that one model is better than another by some amount, even if the evidence may not differ strongly between models. We take this approach as we are only interested in the trend of which models explain the activity best in each source, and our aim is to distinguish between models that may be correlated over time.

In what follows, the input signal for all transformations is the estimated waveform at the tympanic membrane. This waveform was estimated by passing the digital representation of the signal waveform through a digital filter representing the effective frequency response of the earpieces used. This frequency response (called the transfer function) was measured using KEMAR KB0060 and KB0061 artificial ears, mounted in a KEMAR Type 45DA Head Assembly (G.R.A.S. Sound and Vibration, Holte, Denmark). This frequency response, measured as gain relative to 1000 Hz, was estimated to be ([*frequency*:*left gain*:*right gain*]): [125 Hz:–1.5:–0.80], [250 Hz:1.5:1.3], [500 Hz:1.5:2.6], [1000 Hz:0:0], [2000 Hz: 1.6: 0.5], [3000 Hz: –2.0:–0.5] [4000 Hz:–5.6:–3.7], [5000 Hz:–6.4:–5.1], [6000 Hz:–12.3:–13.3].

### MEG and EEG methods and materials

#### Participants and stimuli

##### Participants

Fifteen right-handed participants (7 men, mean age = 24 years, range = 18–30) were recruited. All gave informed consent and were paid for their participation. The study was approved by the Peterborough and Fenland Ethical Committee (UK). For reasons unconnected with the present study, all participants were native speakers of Russian.

##### Stimuli

A single 6 min 40 s acoustic stimulus (a Russian-language BBC radio interview about Colombian coffee) was used. This was later split in the analysis procedure into 400 segments of length 1000 ms. The stimulus was presented at a sampling rate of 44.1 kHz with 16-bit resolution. A visual black fixation cross (itself placed over a video of slowly fluctuating colors) was presented during the audio signal to stop the participant's gaze from wandering.

#### Procedure

Each participant received one practice stimulus lasting 20 s. Subsequent to this, the continuous 6 min 40 s stimulus was presented four times, with instructions to fixate on the cross in the middle of the screen while listening. After each presentation, the participant was asked two simple questions about the content of the stimulus, which they could answer using the button box. Having made a reply, they could rest, playing the next presentation when ready, again using the button box. Presentation of stimuli was controlled with Matlab, using the Psychophysics Toolbox extensions (Brainard, 1997; Pelli, 1997; Kleiner et al., 2007). The stimuli were binaurally presented at ~65 dB SPL via Etymotic Research (Elk Grove Village, Illinois) ER3 earpieces with 2.5 m tubes.

#### EMEG recording

Continuous MEG data were recorded using a 306 channel VectorView system (Elekta-Neuromag, Helsinki, Finland) containing 102 identical sensor triplets (two orthogonal planar gradiometers and one magnetometer) in a hemispherical array situated in a light magnetically-shielded room. The position of the head relative to the sensor array was monitored continuously by four Head-Position Indicator (HPI) coils attached to the scalp. Simultaneous EEG was recorded from 70 Ag-AgCl electrodes placed in an elastic cap (EASYCAP GmbH, Herrsching-Breitbrunn, Germany) according to the 10/20 system, using a nose electrode as reference. Vertical and horizontal EOG were also recorded. All data were sampled at 1 kHz and were band-pass filtered between 0.03 and 330 Hz. A 3-D digitizer (Fastrak Polhemus Inc., Colchester, VA) recorded the locations of the EEG electrodes, the HPI coils and ~50–100 “headpoints” along the scalp, relative to three anatomical fiducials (the nasion and left and right pre-auricular points).

#### Data pre-processing

Static MEG bad channels were detected and excluded from subsequent analyses (MaxFilter version 2, Elektra-Neuromag, Stockholm, Sweden). Compensation for head movements (measured by HPI coils every 200 ms) and a temporal extension of the signal-space separation technique (Taulu et al., 2005) were applied to the MEG data. Static EEG bad channels were visually detected and removed from the analysis (MNE version 2.7, Martinos Center for Biomedical Imaging, Boston, Massachusetts). The EEG data were re-referenced to the average over all channels. The continuous data were low-pass filtered at 100 Hz (zero-phase shift, overlap-add, FIR filtering). The recording was split into 400 epochs of 1000 ms duration. Each epoch included the 200 ms from before the epoch onset and 800 ms after the epoch finished (taken from the previous and subsequent epochs) to allow for the testing of different latencies. Epochs in which the EEG or EOG exceeded 200 μV, or in which the value on any gradiometer channel exceeded 2000 fT/m, were rejected from both EEG and MEG datasets. Epochs for each participant were averaged over all four stimulus repetitions.

#### Source reconstruction

The locations of the cortical current sources were estimated using minimum-norm estimation (MNE) (Hämäläinen and Ilmoniemi, 1994), neuro-anatomically constrained by MRI images obtained using a GRAPPA 3D MPRAGE sequence (TR = 2250 ms; TE = 2.99 ms; flip-angle = 9°; acceleration factor = 2) on a 3T Tim Trio (Siemens, Erlangen, Germany) with 1-mm isotropic voxels. For each participant a representation of their cerebral cortex was constructed using FreeSurfer (Freesurfer 5.3, Martinos Center for Biomedical Imaging, Boston, Massachusetts). The forward model was calculated with a three-layer Boundary Element Model using the outer surface of the scalp and the outer and inner surfaces of the skull identified in the structural MRI. Anatomically-constrained source activation reconstructions at the cortical surface were created by combining MRI, MEG, and EEG data. The MNE representations were downsampled to 10,242 sources per hemisphere, roughly 3 mm apart, to improve computational efficiency. Representations of individual participants were aligned using a spherical morphing technique (Fischl et al., 1999). Source activations for each trial were averaged over participants. We employed a loose-orientation constraint (0.2) to improve the spatial accuracy of localization. Sensitivity to neural sources was improved by calculating a noise covariance matrix based on a 1-s pre-stimulus period. Reflecting the reduced sensitivity of MEG sensors for deeper cortical activity (Hauk et al., 2011), sources located on the cortical medial wall and in subcortical regions were not included in the analyses reported here.

#### Visualization

**Figure 3:**
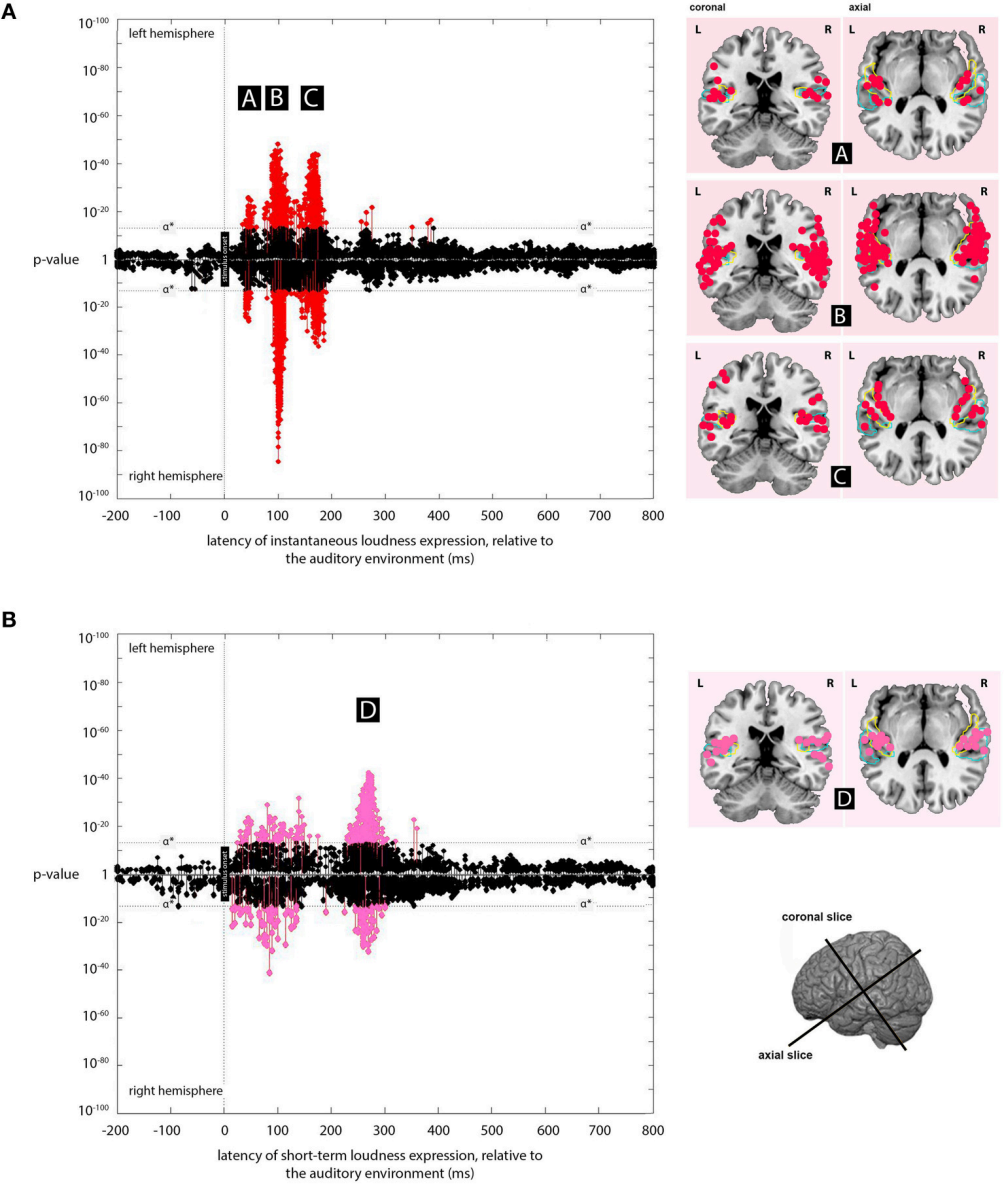
**Expression plots for the *instantaneous loudness* and *short-term loudness* models. (A)** Plot of the expression for the *instantaneous loudness* model across processing lags from -200 to +800 ms, relative to the acoustic signal. Results for the left and right hemispheres are plotted separately, with the right hemisphere plots inverted to allow comparison between hemispheres. The minimum *p*-values at a given source, over all latencies, are marked as “stems.” Stems at or above the stipulated α^*^ value (*p* = 3 × 10^−13^) indicate significant expression of the *instantaneous loudness* model that are not explained better by the *short-term loudness or Hilbert envelope* models. These are marked in red. The cortical locations of significant sources at latencies, **(A)** (45 ms), **(B)** (100 ms), and **(C)** (165 ms) (labeled in black boxes) are indicated on the coronal and axial slices to the right of the plot. **(B)** Plot of the expression for the *short-term loudness* model across processing lags from −200 to +800 ms, relative to the acoustic signal. Again, the cortical locations of significant sources at latency, **(D)** (275 ms) (labeled in a black box) are indicated on the coronal and axial slices to the right of the plot. Both expression plots (with Figure 4) implement models selected so that each source appears only once in the three plots. Probabilistic anatomical landmarks in the slices are provided for planum temporale (light blue) and Heschl's gyrus (yellow) (Rademacher et al., 1992; Morosan et al., 2001; Fischl et al., 2004).

**Figure 4:**
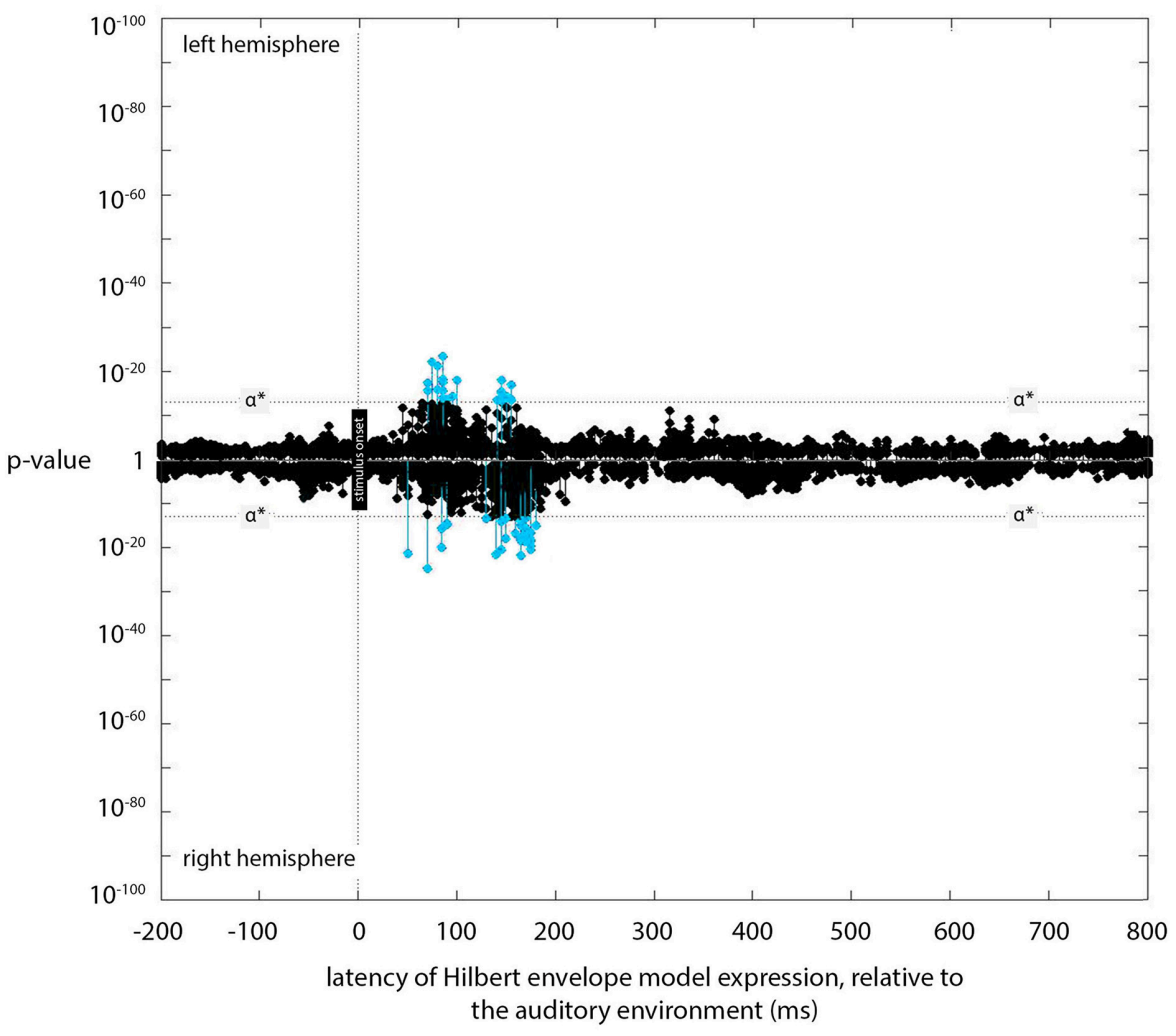
**Expression plot for the *Hilbert envelope* model**. Plot of expression of the *Hilbert envelope* model (across processing lags from −200 to +800 ms, relative to the acoustic signal), showing the latencies of significant expression with the Hilbert model.

The cortical slices in Figures 3, 4 use the visualization software MRIcron (Georgia State Center for Advanced Brain Imaging, Atlanta, Georgia) with results mapped to the high-resolution colin27 brain (Schmahmann et al., 2000). For labeling purposes, two anatomical regions (planum temporale and Heschl's gyrus) were mapped onto the figure using probabilistic atlases (Rademacher et al., 1992; Morosan et al., 2001; Fischl et al., 2004).

## Results

### Instantaneous loudness model

The regions where expression for the instantaneous loudness model was the most significant of the models tested, and below the α^*^ threshold, were located mainly bilaterally with latencies of 45, 100, and 165 ms (Figure 3).

At 45 ms the expression was centered on Heschl's gyrus, while the 100-ms expression was centered on the dorsal lateral sulcus and the dorsal superior temporal sulcus, and the 165-ms expression was centered on medial Heschl's gyrus (to view, see The Kymata Atlas, 2015a). The locations are approximate in all cases, and especially in the 100-ms case, since expression was found in neighboring cortical regions as well. This neighboring expression may be a consequence of the error introduced by the point-spread function inherent in EMEG source localization (see Section Discussion).

### Short-term loudness model

The regions where expression for the short-term loudness model was the most significant of the models tested, and below the α^*^ threshold, were located mainly bilaterally with a latency of 275 ms (Figure 3). This expression was centered on the dorsal lateral sulcus and the dorsal superior temporal sulcus (to view, see The Kymata Atlas, 2015b).

### Hilbert envelope model

Small but significant expression was found for the Hilbert envelope model at 90 and 155 ms (Figure 4). The locations of this expression were similar to the locations of the sources entrained to instantaneous loudness (The Kymata Atlas, 2015c). While significant, the evidence (in terms of *p*-values) for this expression was several orders of magnitude below that for the most significant instantaneous or short-term loudness expression.

## Discussion

As expected, the instantaneous loudness model showed significant bilateral expression in the cortex at 100 ms, in line with the results for this model in Thwaites et al. (2015)^1^. However, in addition to the peak located at ~100 ms, the present data showed distinct peaks at 45 ms and 165 ms. Furthermore, the latencies of the instantaneous loudness expression were more symmetrically distributed than in the previous study. The differences across studies probably reflect the increased accuracy of the present study due to the increase in data (and therefore the reduction in measurement noise). Also, in the present study the frequency response of the earphone was allowed for in calculating the signal at the tympanic membrane, whereas this was not done in the earlier study. As with Thwaites et al. (2015), the present results are broadly in line with the locations and latencies found in other studies of entrainment to models of sound magnitude (e.g., 90 ms in Kubanek et al., 2013; 175/180 ms in (Aiken and Picton, 2008), both in auditory regions). For more information, see Thwaites et al. (2015), including discussion of the difficulties of comparing the apparent cortical entrainment of instantaneous loudness found using the technique of this study and the cortical entrainment to sound magnitude found in other studies.

**Figure 5:**
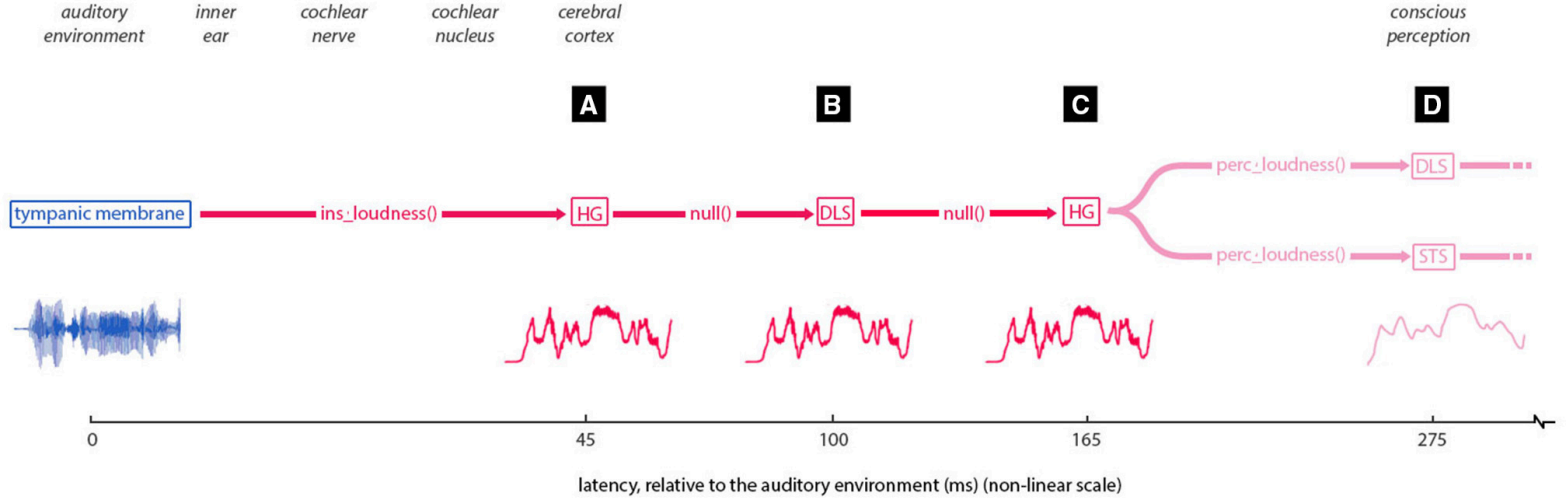
**Implied pathway of loudness information**. Traces one interpretation of the pathway suggested by the findings of this study, with latencies **(A–D)** (labeled in black boxes), corresponding to those same labels in Figures 3A,B. First, the instantaneous loudness transform is applied, with the result of this transformation being entrained in Heschl's gyrus [HG] at 45 ms. This information is then moved or copied (seemingly untransformed) to the dorsal lateral sulcus (at 100-ms latency) and from there back to HG at 165 ms (locations are approximate due to the source localization error). From here, the instantaneous loudness is transformed to the short-term loudness, to be entrained in both the dorsal lateral sulcus and superior temporal sulcus (at 275 ms latency).

It cannot be inferred from these results why instantaneous loudness information is “moved” or “copied” [i.e., with no intervening transform, signaled in Figure 5 by a null() function] from 45 to 100 to 165 ms to different regions of the brain. Storage or preparation for integration with other information, are both possibilities. It may also be the case that these three points of expression are actually the expression of two or more different models, signaling transforms carried out as part of the construction of instantaneous loudness. Such transforms were not tested in this study, but could form an avenue of future research.

The study of Thwaites et al. (2015) only tested for the instantaneous loudness transform and not the short-term loudness transform. As a result, it ascribed entrainment in the STS between 250 and 400 ms to instantaneous loudness. The current study, which assessed entrainment to both the instantaneous loudness and short-term loudness transforms, ascribes this relatively late entrainment to short-term loudness at 275 ms latency. This suggests that the transform between instantaneous loudness and short-term loudness is carried out in the cortex between 165 ms (the last reliable expression of instantaneous loudness) and 275 ms latency.

As an aside, the ascribed entrainment to instantaneous loudness in the previous study between 250 and 400 ms was reported to change position in an anterior direction during this period. No such movement was seen in the current study for short-term loudness.

These findings suggest a set of pathways whereby auditory magnitude information is moved through regions of the cortex (Figure 5). Under this interpretation, the instantaneous loudness transform is applied to auditory information striking the cochlea, with the output of this transformation being entrained in Heschl's gyrus at 45 ms. This information is then moved or copied (seemingly untransformed) to the dorsal lateral sulcus at a latency of 100 ms and from there back to Heschl's gyrus at 165 ms. From here, the instantaneous loudness is transformed to short-term loudness, to be entrained in both the dorsal lateral sulcus and superior temporal sulcus at 275 ms latency.

Inevitably, the cortical locations of the expressions must be treated with caution. The inherent insolvability of the inverse problem during EMEG source reconstruction (Grave de Peralta-Menendez et al., 1996; Grave de Peralta-Menendez and Gonzalez-Andino, 1998) means that significant “point spread” of localization data was present; improvements in source reconstruction (through the gathering of more data or improved inverse techniques) may ameliorate this problem in the future.

Source estimation error is also likely to account for the fact that a few sources showed the most significant entrainment to the Hilbert envelope model. Although the number of sources showing significant entrainment to the Hilbert envelope model was much lower than for the instantaneous or short-term loudness models, and the average *p*-values were much higher than the average *p*-values for instantaneous or short-term loudness (and thus not included in Figure 5), the behavior of these few sources was better explained by the Hilbert envelope model. Yet we know that the Hilbert envelope model is unrealistic, as it takes no account of the physical transformations occurring between the sound source and the cochlea or of the physiological transformations occurring in the cochlea. Likewise, some sources showed significant entrainment to short-term loudness before 45 ms; since the short-term loudness transform must have a longer latency than instantaneous loudness, significant expression in these sources may well be due to estimation error or to the strong grouping of most of the entrained sources (temporally and spatially). The only way of assessing whether these unexpected effects are or are not due to measurement error is to obtain better/more data and to reduce source estimation error.

The results presented here cannot support or disprove the suggestion that “loudness” may be constructed subsequent to processing in the IC (Röhl and Uppenkamp, 2012), as we were unable to measure activity in the IC due to the limitations of EMEG recordings. In any case, the result of our study and theirs are difficult to compare, since they looked for relationships between brain activity and categorical judgments of loudness rather than using a model to generate loudness predictions.

Distinguishing between the processing of instantaneous loudness, which is unavailable for conscious perception (and thus difficult to test behaviorally), and short-term loudness, which is perceivable, is challenging. Evidence supporting the idea that instantaneous loudness is a prior transform to short-term loudness can be found in studies of the perception of sounds that are sinusoidally amplitude modulated at various rates. If the rate is low, say 4 Hz, and the modulation depth is high, then distinct loudness fluctuations are heard (Moore et al., 1999). Correspondingly, the loudness model of Glasberg and Moore (2002) predicts distinct fluctuations in short-term loudness for such stimuli. However, if the rate is increased to, say, 100 Hz, the sound quality is heard as “rough,” but the loudness appears to be constant (Moore et al., 1999). Correspondingly, the loudness model of Glasberg and Moore (2002) predicts almost no fluctuations in short-term loudness for such stimuli. Nevertheless, the amplitude fluctuations can be heard; the modulated sound is perceived as different from an unmodulated sound of the same loudness. Indeed amplitude fluctuations can be detected for rates up to about 800–1000 Hz (Kohlrausch et al., 2000). These findings suggest that fluctuations in instantaneous loudness contribute to the perception of roughness and to the detection of amplitude modulation, but that the perception of short-term loudness depends on temporal integration of the instantaneous loudness. The results of the current study support this view.

### Overview

The results from this study suggest that the instantaneous loudness transform of incoming sound happens before 45 ms latency, and entrainment to the instantaneous loudness transform occurs in different regions of the cortex at latencies of 45, 100, and 165 ms (bilaterally, in HG, DLS, and HG, respectively). Subsequent to this, the short-term loudness transform is applied, with entrainment primarily at 275 ms, bilaterally in DLS and STS. The locations of this entrainment are only approximate due to the inherent error in source estimation of EMEG data. More work is needed to improve the accuracy of these reconstructions in order to improve the certainty of these locations.

## Author contributions

AT and INS devised the methods. BG and BM developed the hypotheses. AT collected the data. WMW gave guidance and advice. AT and BM drafted the paper. All contributed to the final draft.

### Conflict of interest statement

The authors declare that the research was conducted in the absence of any commercial or financial relationships that could be construed as a potential conflict of interest.

## Footnotes

^1^Readers should remember that latencies here refer to the period between the auditory information striking the tympanic membrane and significant entrainment to this information in the cortex; this definition of latency is not equivalent to latencies reported in, for instance, “evoked response potential” studies (P1, N1, P3 etc.) which refer to the period between the onset of a stimulus and a spike (or otherwise) in the measured neural activity.
